# Incidence of completed suicide and suicide attempts in a global prospective study of Huntington's disease

**DOI:** 10.1192/bjo.2021.969

**Published:** 2021-08-31

**Authors:** Erik van Duijn, A. Raquel Fernandes, Daisy Abreu, Jennifer J. Ware, Eileen Neacy, Cristina Sampaio

**Affiliations:** Department of Psychiatry, Leiden University Medical Center, The Netherlands and Huntington Center Topaz Overduin, The Netherlands; Association for Research and Development of the Faculty of Medicine, University of Lisbon, Portugal; Association for Research and Development of the Faculty of Medicine, University of Lisbon, Portugal; CHDI Management/CHDI Foundation, New Jersey, USA; CHDI Management/CHDI Foundation, New Jersey, USA; CHDI Management/CHDI Foundation, New Jersey, USA

**Keywords:** Suicide, clinical neurology, self-harm, epidemiology, Huntington's disease

## Abstract

**Background:**

Risk of death from suicide in Huntington's disease is notably elevated relative to that in the general population, although the incidence within HD populations has not been precisely defined. Robust incidence estimates of suicidal behavior can serve as references for HD therapeutic research and post-marketing surveillance to help evaluate the suicidality risk of novel therapeutics.

**Aims:**

To estimate the incidence rate of completed suicide and suicide attempt in the global, prospective HD cohort study Enroll-HD that records these events per protocol.

**Method:**

A total of 20 912 participants were available for analysis (HD gene-expansion carriers (HDGECs) *n* = 15 924; non-HDGECs n = 4988) representing a collective observation period of 53 390 participant-years. Each observed event was subject to clinical review and evaluation. We generated incidence rates (events per 100 000 person-years) for suicides and suicide attempts using all available data, as well as by year of study and geographical region. Proportionate mortality statistics for suicide and respective 95% confidence intervals were also generated.

**Results:**

The overall incidence rate of suicide in HDGECs was 72 per 100 000 person-years, and 8 per 100 000 person-years in non-HDGECs. Proportionate mortality attributable to suicide in HDGECs was 4.6%. For suicide attempts, the global overall incidence rate observed in HDGECs was 306–375 per 100 000 person-years, and 23–38 per 100 000 person-years in non-HDGECs.

**Conclusions:**

The incidence estimates calculated here can be used as a reference to help evaluate drug safety and may also be useful in assessing progress in clinical care for HDGECs once therapeutic interventions become widely available.

Huntington's disease is a progressive neurodegenerative disease for which there is no current effective treatment, except for symptomatic treatments to manage psychiatric and movement symptoms. In Huntington's disease, the risk of death by suicide has been estimated to be two to seven times greater than that observed in the general population.^1–3^ Suicide is the third most common cause of all deaths among patients with manifest Huntington's disease (after pneumonia and other infections), accounting for up to 6.6% of deaths,^1^ and estimates of lifetime prevalence of suicidal ideation among patients with Huntington's disease are as high as 20%.^2,4,5^ A previous study showed that death by suicide among people with neurological disorders occurs more often in patients with amyotrophic lateral sclerosis or Huntington's disease than in patients with multiple sclerosis, head injury, stroke or epilepsy.^6^ This increased risk of suicidal behaviour frames the clinical management of Huntington's disease. Huntington's disease is a neurodegenerative disorder characterised by progressive motor dysfunction, neuropsychiatric symptoms and neurocognitive deterioration. Huntington's disease is an autosomal dominant hereditary disease caused by an expanded CAG trinucleotide repeat in the huntingtin gene on chromosome 4.^7^ In most Huntington's disease gene expansion carriers (HDGECs), clinical signs and symptoms become manifest in the fourth or fifth decade of their lives. with a survival time of around 15 years after clinical motor diagnosis onset.^8^ Common neuropsychiatric symptoms are depressed mood, irritability, apathy and obsessive–compulsive behaviours that can present in all stages of the disease and may precede motor signs.^9^ The increased occurrence of neuropsychiatric symptoms and neurocognitive deficits like depressed mood, impulsivity, impaired abstract thinking and impaired problem-solving capacity are risk factors for suicide.^10^ Also, when facing the prospect of onset and steady progression of a disease for which there is currently no effective treatment, patients may feel entrapped and see suicide as an escape. The only two drugs approved for Huntington's disease (tetrabenazine and deutetrabenazine) are to treat chorea, and both are associated with an increased risk of depression and suicide,^11^ although there is disagreement as to whether tetrabenazine increases suicide risk.^12–14^ Although Huntington's disease is associated with an increased risk of suicide, the precise incidence in this population is not defined. Robust incidence estimates of suicide and suicide attempts in the HDGEC population are needed as a reliable reference for ongoing and future Huntington's disease therapeutic research and post-marketing surveillance, to help evaluate the suicidality risk of novel Huntington's disease therapeutics. Here, we aimed to estimate the incidence rate of suicide attempts and suicide in a global, prospective Huntington's disease cohort study, to provide robust references for Huntington's disease therapeutic development.

## Method

### Study population

Enroll-HD (https://www.enroll-hd.org/) is a prospective cohort study and global clinical research platform designed to facilitate clinical research in Huntington's disease.^15^^,^^16^ Enroll-HD encompasses over 20 000 participants from approximately 160 active sites located in 19 countries across North America, Latin America, Europe and Australasia. Data are collected from participants annually, and are monitored for quality and accuracy using a risk-based monitoring approach. The data for this study were extracted from the Enroll-HD electronic data capture system database on 1 May 2019. Participants were classified as HDGECs or non-HDGECs based on CAG-repeat length, as determined in a central laboratory (Biorep Technologies, Inc). Individuals with a CAG-repeat length of ≥36 were classified as HDGECs (*n* = 15 924), and those with a CAG-repeat length of ≤35 were classified as non-HDGECs (*n* = 4988). The latter group comprised genotype-negative individuals and family members or individuals not related by blood to HDGECs. Individuals with unknown CAG-repeat length (*n* = 327) were excluded from analyses. This resulted in 20 912 participants who were available for analysis.

### Observation period

Enroll-HD baseline visit date (i.e. study entry) was considered as the beginning of the observation period for each participant. For participants who died or discontinued the study, date of death as reported on the Mortality form (or Reportable Event form in absence of the Mortality form) or date of Premature End form completion was used to define the end of the observation period. Participants who had not died nor formally discontinued, but had missed two consecutive annual visits (operationally defined as the elapsing of >2 years and 3 months since the due date of the previous in-person visit) were considered ‘lost to follow-up’; the date 2.25 years after the due date of their last Enroll-HD visit was used to define the end of the time-at-risk period. For all other (i.e. active) participants within the 2.25-year window after their last visit at the time of data cut, the date of the data cut (1 May 2019) was used to define the end of the time-at-risk period.

Pursuant to the above definition, the total observation period for HDGECs comprised 40 242 person-years, and for non-HDGECs comprised 13 148 person-years. Incidence rates were calculated per 100 000 person-years, according to the following formula: (observed events (within indicated time period) / person-years (within indicated time period)) × 100 000.

### Variables

**Fig. 1:**
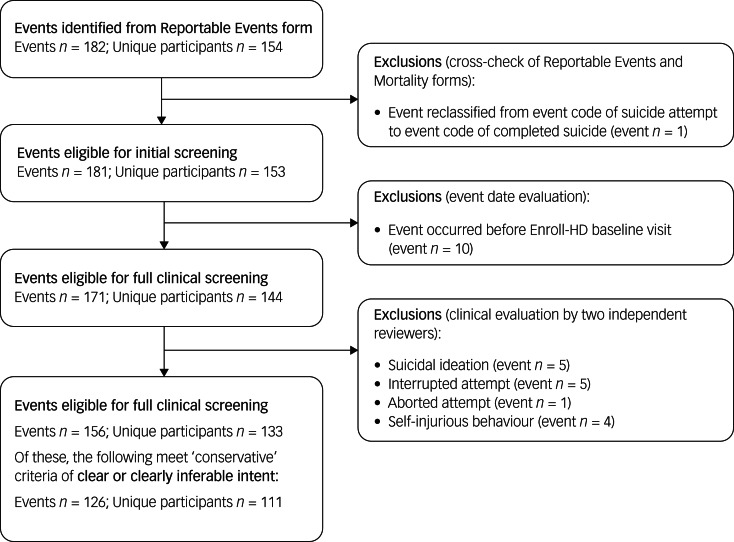
Event flowchart: suicide attempts.

**Fig. 2:**
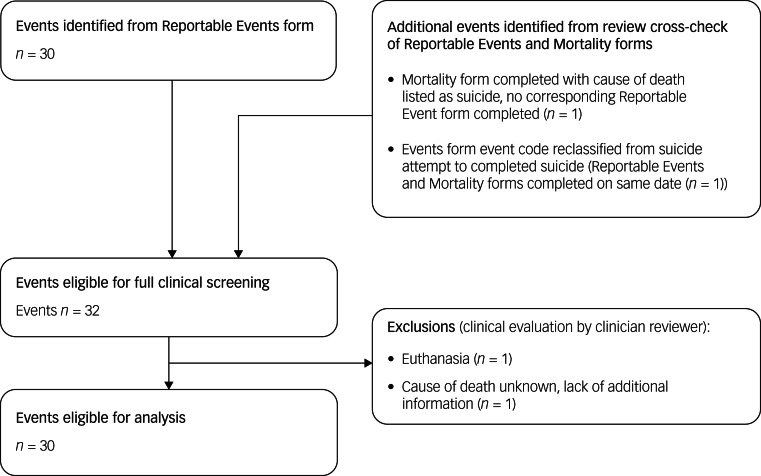
Event flowchart: completed suicides.

Data on ‘reportable events’ are recorded per Enroll-HD protocol, and comprise suicide attempt, completed suicide, mental health event requiring hospital admission, and death (other than suicide). Such events are recorded on a Reportable Events form, which can be completed at any time during or outside of a standard Enroll-HD visit. This form was the primary source for identifying suicide attempts and completed suicides. All Reportable Events forms are sent to the Data and Safety Monitoring Committee to assess site compliance to screening procedures and assessment protocols. Mortality forms, which are completed in the event of death, were used as a secondary source for identifying missed events. A quality-control process was used to screen and categorise identified events (Figs 1 and 2). Events dated before the Enroll-HD baseline visit date were excluded. Remaining events were then subject to clinical evaluation, as described below.

#### Suicide attempts

**Fig. 3:**
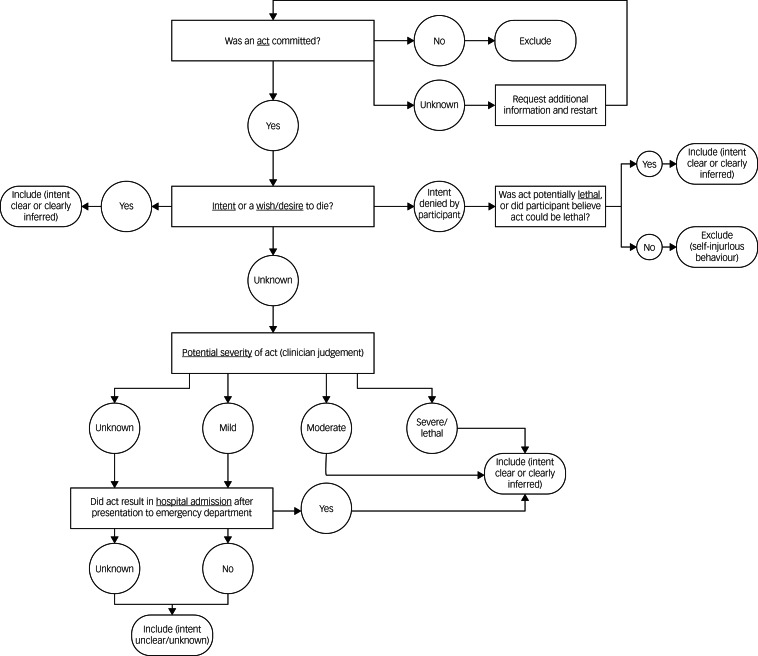
Suicide attempt evaluation and classification decision tree.

All events were subject to clinical evaluation by two independent reviewers (E.v.D. and J.J.W.). A decision tree, based on the Columbia-Suicide Severity Rating Scale (C-SSRS) definition of an ‘actual attempt’,^17^ was created (Fig. 3) to evaluate event narratives and include or exclude cases accordingly and consistently. Excluded events were categorised as follows: self-injurious behaviour (*n* = 4), interrupted attempt (*n* = 5), aborted attempt (*n* = 1) and suicidal ideation (*n* = 6). The National Toxicology Information Center (The Netherlands) was consulted to evaluate potential severity of drug overdoses. As in some cases the intent of the suicidal behaviour was not clear or clearly inferable, or inadequate information was provided to allow categorisation of an event, additional information was requested from Enroll-HD sites. Where additional information could not be provided, the event was included under the ‘liberal’ estimate, to avoid a possible underestimation of the incidence of suicide attempts. A ‘conservative’ estimate was generated based on conclusively reported cases only.

#### Completed suicides

All reports of completed suicides were subject to clinical evaluation by one rater (E.v.D.). Narratives provided on the Reportable Events form were evaluated, alongside cause of death and narratives provided on associated Mortality forms. Additional information was requested for one case to assist in decision-making. Clinical evaluation resulted in the exclusion of two cases for reasons of euthanasia (*n* = 1) and cause of death listed as unknown with lack of information (*n* = 1).

#### Sociodemographic and clinical characteristics

Sociodemographic and clinical variables were used to characterise participants (Table 1). Clinical variables included psychiatric measures (e.g. prior suicidal ideation per C-SSRS; depression, anxiety, apathy and irritability subscale scores per the short version of the Problem Behavior Assessment) and clinical assessments encompassing other Huntington's disease-specific disease domains: motor (e.g. Unified Huntington's Disease Rating Scale (UHDRS) total motor score (TMS)), function (e.g. UHDRS total functional capacity (TFC)) and cognition (e.g. Symbol Digit Modality Test (SDMT)). To characterise the full sample, variable values ascertained at the baseline visit were used. To characterise participants who experienced an event, variable values were ascertained at the Enroll-HD visit before the event. Only age and CAG-age product (CAP) score were determined based on age on event date. CAP score, calculated per the following formula, is indicative of cumulative exposure to mutant huntingtin (akin to ‘pack-years’ for assessing tobacco exposure in smokers):^18^
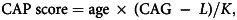
where *L* = 30 and *K* = 6.49.

**Table 1:** Participant characterisation

	Full sample				Attempted suicide (liberal definition)				Completed suicide			
	HDGECs (*n* = 15 924)		Non-HDGECs (*n* = 4988)		HDGECs (obseved events *n* = 151; unique participants *n* = 129)		Non-HDGECs (observed events *n* = 5; unique participants *n* = 4)		HDGECs (*n* = 29)		Non-HDGECs (*n* = 1)	
	*n* = Missing	*n* = Missing	*n* = Missing	*n* = Missing	*n* = Missing	*n* = Missing
Sociodemographic measures												
Age, median (IQR)	1	49.0 (38.0–59.0)	1	48.0 (35.0–59.0)	0	48.0 (41.0–56.0)	0	53.0 (42.0–53.0)	0	55.0 (51.0–64.0)	0	23.0 (23.0–23.0)
Gender (female), *n* (%)	1	8673 (54.5%)	1	3049 (61.1%)	0	87 (57.6%)	0	2 (40.0%)	0	10 (34.5%)	0	0 (0.0%)
Partnered (no, not married or partnered), *n* (%)	75	5882 (37.1%)	19	1289 (25.9%)	69	33 (40.2%)	2	1 (33.3%)	17	2 (16.7%)	0	1 (100.0%)
Huntington's disease-specific clinical measures												
Disease status (manifest), *n* (%)	262	26 694 (67.8%)			0	122 (80.8%)		–	0	27 (93.1%)		–
CAP score, median (IQR)	1	101.7 (81.4–114.3)		–	0	104.8 (93.9–115.7)		–	0	111.9 (105.7–118.6)		–
TFC score, median (IQR)	143	11.0 (7.0–13.0)	29	13.0 (13.0–13.0)	0	8.0 (6.5–11.0)	0	13.0 (13.0–13.0)	0	9.0 (7.0–11.0)	0	13.0 (13.0–13.0)
TMS score, median (IQR)	222	23.0 (5.0–43.0)	43	0.0 (0.0–2.0)	0	30.0 (19.0–41.5)	0	8.0 (0.0–17.0)	0	38.0 (22.0–48.0)	0	0.0 (0.0–0.0)
SDMT score, median (IQR)	1105	30.0 (18.0–46.0)	70	51.0 (43.0–58.0)	6	21.0 (15.0–32.0)	0	46.0 (35.0–46.0)	0	20.0 (16.0–28.0)	0	60.0 (60.0–60.0)
Psychiatric measures												
Prior suicidal ideation (C-SSRS, yes), *n* (%)	8856	2489 (35.2%)	2972	429 (21.3%)	52	59 (59.6%)	2	3 (100.0%)	8	6 (28.6%)	0	1 (100.0%)
Depression (PBA-s score), median (IQR)	270	0.0 (0.0–4.0)	32	0.0 (0.0–2.0)	1	4.0 (0.0–9.0)	0	2.0 (2.0–9.0)	0	1.0 (0.0–4.0)	0	4.0 (4.0–4.0)
Anxiety (PBA-s score), median (IQR)	277	1.0 (0.0–4.0)	34	0.0 (0.0–3.0)	1	4.0 (0.0–6.0)	0	4.0 (2.0–4.0)	0	0.0 (0.0–4.0)	0	0.0 (0.0–0.0)
Apathy (PBA-s score), median (IQR)	295	0.0 (0.0–4.0)	35	0.0 (0.0–0.0)	1	4.0 (0.0–8.0)	0	3.0 (0.0–4.0)	0	2.0 (0.0–3.0)	0	6.0 (6.0–6.0)
Irritability (PBA-s score), median (IQR)	197	1.0 (0.0–4.0)	34	0.0 (0.0–1.0)	1	2.0 (0.0–6.0)	0	0.0 (0.0–4.0)	0	0.0 (0.0–4.0)	0	6.0 (6.0–6.0)
Antidepressant use, lifetime (yes), *n* (%)	1043	8146 (54.7%)	292	928 (19.8%)	3	115 (91.3%)”	0	4 (100.0%)	2	18 (66.7%)	0	0 (0.0%)
Smoking, currently (yes), *n* (%)	79	4035 (25.5%)	23	999 (20.1%)	1	45 (30.0%)	0	1 (100.0%)	0	10 (34.5%)	0	1 (100.0%)
Drug use, currently (yes), *n* (%)	80	610 (3.9%)	25	287 (5.8%)	1	51 (34.2%)	0	4 (80.0%)	0	1 (3.4%	0	0 (0.0%)
Alcohol use, currently (yes), *n* (%)	81	7126 (45.0%)	25	2665 (53.7%)	2	4 (13.8%)	0	0 (0.0%)	0	12 (41.4%)	0	1 (100.0%)

HDGECs, Huntington's disease gene expansion carriers; IQR, interquartile range; CAP, CAG-age product; TFC, total functional capacity; TMS, total motor score; SDMT, Symbol Digit Modality Test; C-SSRS, Columbia-Suicide Severity Rating Scale; PBA-s, Problem Behaviors Assessment - Short.

As 18 participants had more than one suicide attempt during the observation period, the number of unique events and the number of unique participants differs. For characterisation of suicide attempts, descriptive statistics were generated based on the number of unique events.

#### Deaths (all cause)

To determine proportionate mortality attributable to suicide in Enroll-HD, data on deaths (all causes) were required. The Mortality form (completed in the event of participant death) and the Reportable Events form were the reference sources for this variable.

The research participants who contribute their data and samples to Enroll-HD give voluntary written informed consent in accordance with General Data Protection Regulation (European Union). The informed consent was purposefully developed to ensure research use and protect the confidentiality and privacy of the contributors. Institutional review board/independent ethics committee approval, consistent with local regulations, has been obtained by each Enroll-HD site. The authors assert that all procedures contributing to this work comply with the ethical standards of the relevant national and institutional committees on human experimentation and with the Helsinki Declaration of 1975, as revised in 2008.

### Statistical methods

Analyses were limited to generation of descriptive statistics. Incidence/event rate statistics were generated for suicide attempts and completed suicides, using all available data. Incidence statistics are also presented by year of study and region. Proportionate mortality statistics and respective 95% confidence interval for suicide were also generated. The 95% confidence interval estimation for the binomial proportions was based on the bootstrap percentile-*t* method as described by Mantalos and Zografos.^19^ Strengthening the Reporting of Observational studies in Epidemiology guidelines were followed in reporting. All descriptive statistics are presented by gene status.

Missing or incomplete event dates were imputed according to the rules outlined in Supplementary Appendix 1 available at https://doi.org/10.1192/bjo.2021.969. Additional information regarding event narratives was requested from study sites where insufficient information was provided to include or exclude or categorise events. Missing data for other variables were not imputed. Missing data points by variable are indicated in Table 1. Statistical analyses were performed with R version 3.4.2 for Windows (R Core Team; Vienna, Austria; https://www.R-project.org/).

## Results

### Frequency statistics

**Fig. 4:**
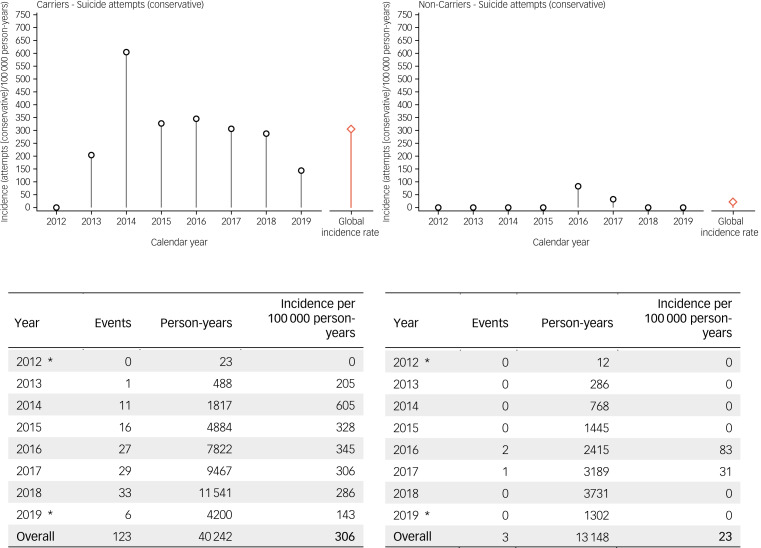
Incidence rates of suicide attempts in Enroll-HD (conservative estimate). left panel: HDGECs; right panel: non-HDGECs; incidence rates are displayed by year, and overall (red bar). *Period of observation began 25 July 2012 and ended 1 May 2019. HDGECs, Huntington's disease gene expansion carriers.

**Fig. 5:**
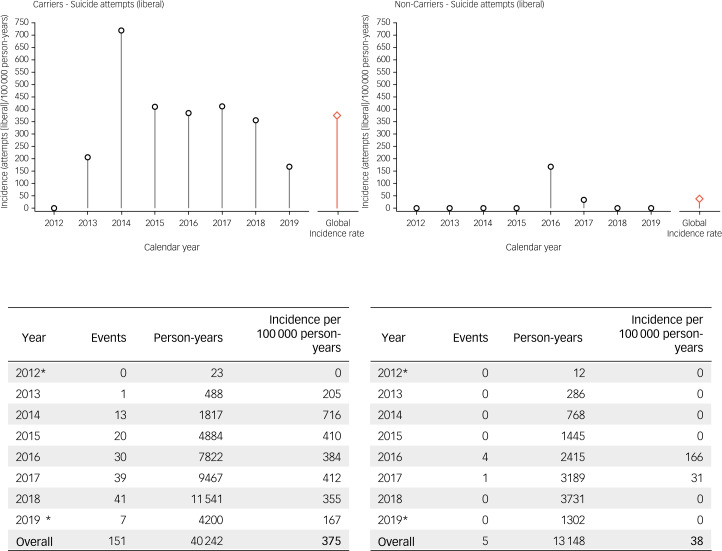
Incidence rates of suicide attempts in Enroll-HD (liberal estimate). left : HDGECs; right: non-HDGECs; incidence rates are displayed by year, and overall (red bar). *Period of observation began 25 July 2012 and ended 1 May 2019. HDGECs, Huntington's disease gene expansion carriers.

Of the 20 912 Enroll-HD participants considered, representing a total of 53 390 participant-years, a total of 30 completed suicides were observed; 29 were observed in HDGECs and one was observed in a non-HDGEC (Table 1 and Fig. 2). For suicide attempts, a total of 126 events with clear or clearly inferable intent were observed in 111 unique participants (conservative estimate); 123 of these events were observed in HDGECs and three in non-HDGECs (Figs 1 and 4). The total number of suicide attempts increased to 156 events in 133 unique participants when inclusion of events was expanded to incorporate events in which intent was unclear (liberal estimate); 151 of these events were observed in HDGECs and five in non-HDGECs (Table 1 and Figs 1 and 5).

Multiple suicide attempts were observed in 18 participants. Fourteen participants reported two attempts, three reported three attempts and one reported four attempts. Of the 30 participants who died by suicide, two had reported a prior suicide attempt. The median length of time between event date and date of Reportable Event form completion for completed suicides and suicide attempts was 28 days (interquartile range 3–155). A total of 659 deaths (all causes, including suicide) were observed in our analysis sample over the time period examined; 637 in HDGECs and 22 in non-HDGECs.

#### Suicide attempts

Incidence rates of suicide attempts in Enroll-HD, using the conservative and liberal estimate, respectively, are presented by gene status and year (2012–2019) in Figs 4 and 5, and by gene status and region (North America, Europe, Latin America, Australasia) in Tables 2 and 3. For suicide attempts where intent was clear or clearly inferable (i.e. conservative estimate), the global overall incidence rate observed in HDGECs was 306 per 100 000 person-years (Fig. 4). The analogous figure in non-HDGECs was 23 per 100 000 person-years (Fig. 4). When all suicide attempts were considered, including events where intent was not clear or clearly inferable (i.e. liberal estimate), the global overall incidence rate in HDGECs was 375 per 100 000 person-years, and 38 per 100 000 person-years in non-HDGECs (Fig. 5).

**Table 2:** Comparative incidence rates for suicide attempts (liberal) in Enroll-HD, by geographical region

Region	Gene status	Events	*n*	Person-years	Overall incidence per 100 000 person-years
North America	HDGEC	38	4865	13 860	274
	Non-HDGEC	4	2388	7320	55
Europe	HDGEC	97	10 268	24 213	401
	Non-HDGEC	0	2349	5197	0
Latin America	HDGEC	3	172	374	802
	Non-HDGEC	1	80	177	565
Australasia	HDGEC	13	619	1795	724
	Non-HDGEC	0	171	454	0

; HDGECs, Huntington's disease gene expansion carriers.

**Table 3:** Comparative incidence rates for suicide attempts (conservative) in Enroll-HD, by geographical region

Region	Gene status	Events	*n*	Person-years	Overall incidence per 100 000 person-years
North America	HDGEC	36	4865	13 860	260
	Non-HDGEC	2	2388	7320	27
Europe	HDGEC	75	10 268	24 213	310
	Non-HDGEC	0	2349	5197	0
Latin America	HDGEC	3	172	374	802
	Non-HDGEC	1	80	177	565
Australasia	HDGEC	9	619	1795	501
	Non-HDGEC	0	171	454	0

HDGECs, Huntington's disease gene expansion carriers.

#### Completed suicides

**Fig. 6:**
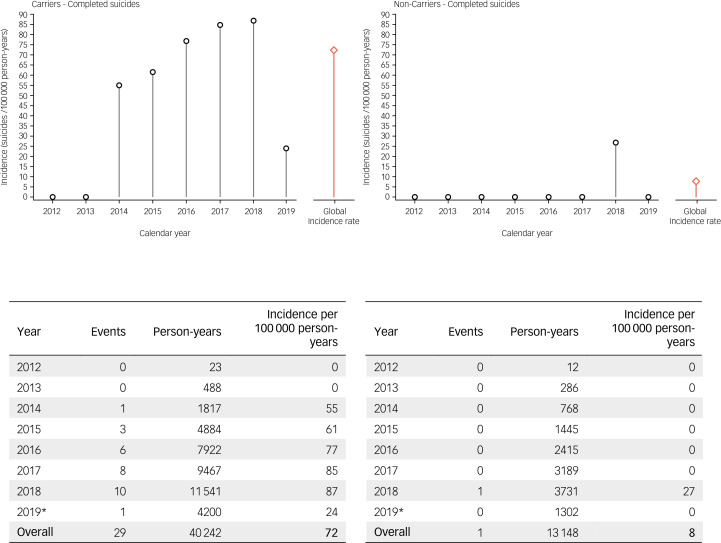
Incidence rates of completed suicide in Enroll-HD. left: HDGECs; right: non-HDGECs; incidence rates are displayed by year, and overall (red bar). *Period of observation began 25 July 2012 and ended 1 May 2019. HDGECs, Huntington's disease gene expansion carriers.

Suicide rates in Enroll-HD are presented by gene status and year in Fig. 6, and by gene status and region in Table 4. Considering all available data, the overall incidence rate of suicide was 72 per 100 000 person-years in HDGECs, and 8 per 100 000 person-years in non-HDGECs. . The proportionate mortality attributable to suicide in Enroll-HD is 4.6% (29 out of 637) in HDGECs (95% CI 2.84–5.87%), and 4.5% (1 out of 22) in non-HDGECs (95% CI 0–13.51%).

**Table 4:** Comparative incidence rates for completed suicides in Enroll-HD, by geographical region

Region	Gene status	Events	*n*	Person-years	Overall incidence per 100 000 person-years
North America	HDGEC	8	4865	13 860	58
	Non-HDGEC	1	2388	7320	14
Europe	HDGEC	17	10 268	24 213	70
	Non-HDGEC	0	2349	5197	0
Latin America	HDGEC	1	172	374	267
	Non-HDGEC	0	80	177	0
Australasia	HDGEC	3	619	1795	167
	Non-HDGEC	0	171	454	0

HDGECs, Huntington's disease gene expansion carriers.

### Sociodemographic and clinical characteristics

Sociodemographic and clinical characteristics of participants who attempted or completed suicide are provided in Table 1.

#### Suicide attempts

Of the 151 suicide attempts observed in HDGECs (liberal estimate), 58% (*n* = 87) were observed in females, and median age at event was 48 years. With regards to disease stage and severity, 81% (*n* = 122) of events were observed in manifest individuals, with a median CAP score at event of 105. Median TFC score at event was 8, median TMS at event was 30 and median SDMT score was 21. Prior suicidal ideation, as assessed by the C-SSRS, had been reported by 60% (*n* = 59).

#### Completed suicides

Of those HDGECs who died by suicide, 35% (*n* = 10) were female, and median age of event was 55 years. With regards to disease stage and severity, 93% (*n* = 27) were manifest, with a median CAP score of 112. Median TFC score was 9, median TMS was 38 and median SDMT score was 20. Prior suicidal ideation, as assessed by the C-SSRS, had been reported by only 29% (*n* = 6).

## Discussion

For the first time, robust estimates of the incidence of suicide attempts and completed suicides are provided in a large and globally representative Huntington's disease cohort, encompassing a broad disease-stage spectrum. Our results confirm the elevated occurrence of completed suicides and suicide attempts in HDGECs that were previously reported in smaller and mostly retrospective studies. Besides their relevance in clinical practice, the incidence estimates calculated here will be a useful reference to evaluate therapeutic safety, since suicidality is an event of interest in the development of any drug targeting the central nervous system, as per Food and Drug Administration guidance.^20^ Completed suicides and even suicide attempts in the context of clinical trials are relatively scarce and therefore difficult to evaluate, so knowing the estimated suicide/suicide attempt incidence in a representative cohort is important.

We provide both conservative and liberal estimates of suicide attempt incidence rate in Enroll-HD because the intent of the suicidal act was not always clear from event narratives. When only the conservative approach was used, this may have underestimated the incidence of suicide attempts. Including events in which the intent is unclear or questionable in our calculations is in line with clinical practice. The estimates from both approaches resulted in a range for the annual incidence of 306–375 per 100 000 person-years in HDGECs, and 23–38 per 100 000 person-years in non-HDGECs, where the lower values represent estimates from conservative approaches. Regional differences were also noted, although the limited number of events in certain regions restricts meaningful comparison. Comparable incidence estimates of suicide attempts in the general population are not known, but the World Health Organization has estimated that the 12-month prevalence of suicide attempts is approximately 0.3%.^21^

Although many prior studies have reported on suicidality in Huntington's disease with regards to suicidal *ideation* and risk factors for suicidal ideation,^4,22,23^ there is limited data on suicide attempts and completed suicides in Huntington's disease. In the neurobiological predictors of Huntington's disease study (PREDICT-HD),^24^ an observational study of premanifest HDGECs, 13 suicide attempts over 2718 person-years were reported, which is equal to a suicide attempt incidence of 478 per 100 000 person-years (Table 5), which is higher than our HDGEC estimate. In a randomized, double-blind, placebo-controlled trial of coenzyme Q10 in Huntington's disease (2CARE),^25^ a clinical trial conducted in patients with early-manifest Huntington's disease, 22 suicide attempts were reported over <3045 person-years, which is equivalent to at least 722 per 100 000 person-years. Data from a phase 2, dose-finding, randomized, parallel-group, double-blind, placebo-controlled study, evaluating the safety and efficacy of Pridopidine 45 mg, 67.5 mg, 90 mg, and 112.5 mg twice-daily versus placebo for symptomatic treatment in patients with Huntington's disease (PRIDE-HD)^26^, a clinical trial that enrolled manifest patients, reported four suicide attempts over <204 person-years regardless of treatment group considered, which is equivalent to at least 1961 per 100 000 person-years, although the limited sample size and limited prospective follow-up period calls into question the accuracy of this estimate.

**Table 5:** Comparative incidence rates for suicide attempts in HDGECs

Study	Disease status of sample	Events	*n*	Person-years	Overall incidence per 100 000 person-years
Enroll-HD (liberal)	Premanifest and manifest	151	15 924	40 242	375
Enroll-HD (conservative)	Premanifest and manifest	123	15 924	40 242	306
PREDICT-HD	Premanifest	13	735	2718	478
2CARE	Early manifest	22	609	<3045	>722
PRIDE-HD	Manifest	4	408	<204	>1961

HDGECs, Huntington's disease gene expansion carriers.

The incidence rate of completed suicides among HDGECs in Enroll-HD is 72 per 100 000 person-years, which is about four to six times the incidence rate of 11.9 and 21.1 suicides per 100 000 person-years for the general population in the UK and USA, respectively.^27^ This is notably lower than the incidence rate of suicide of 203.6 per 100 000 person-years observed in a Danish cohort of symptomatic patients with Huntington's disease, although this estimate was based on a much smaller cohort of patients (*n* = 1228),^6^ and the suicide rate in the general population in Denmark has historically been high.^28^ We found a slightly higher incidence of completed suicide among HDGECs in Enroll-HD in Europe (70 per 100 000 person-years) compared with North America (58 per 100 000 person-years); however, no conclusions can be drawn because of the small numbers after regional stratification (Table 4). The incidence rate of completed suicide in non-HDGECs in Enroll-HD is 8 per 100 000 person-years, which is close to the global age-standardised completed suicide rate of 10.5 per 100 000.^27^

In the PREDICT-HD study,^24^ a completed suicide rate of 37 per 100 000 person-years was reported (one event over 2718 person-years), and in the 2CARE study, 164 per 100 000 person-years (five events over <3045 person-years) was reported,^25^ whereas no completed suicides were reported in the PRIDE-HD study (Table 6).^26^ In the PREDICT-HD study, participants were strictly defined as premanifest individuals at the time of inclusion, and participants are therefore in a less advanced disease stage compared with the Enroll-HD population, whereas in Enroll-HD, the majority of HDGECs were manifest at baseline (68%; see Table 1). Clinical trial populations (2CARE, PRIDE-HD) are distinct from natural cohorts because participants with severe mental health problems or with an *a priori* increased suicide risk are often excluded, which likely decreases the risk of suicidality events in trial populations. On the other hand, the strict monitoring of participants in trials and the higher frequency of visits may inflate the reporting.

**Table 6:** Comparative incidence rates for completed suicides in HDGECs

Study	Disease status of sample	Events	*n*	Person-years	Overall incidence per 100 000 person-years
Enroll-HD	Premanifest and manifest	29	15 924	40 242	72
PREDICT	Premanifest	1	735	2718	37
2CARE	Early manifest	5	609	<3045	164
PRIDE-HD	Manifest	0	408	<204	0

HDGECs, Huntington's disease gene expansion carriers.

We found that 4.6% (95% CI 2.84–5.87%) of all reported HDGEC deaths in Enroll-HD were from suicide, which is consistent with an analysis of death certificates of patients with manifest Huntington's disease in Denmark (5.6%)^29^ and the 6.6% of patients with manifest Huntington's disease that was previously assessed in REGISTRY, the European observational study that has been incorporated into Enroll-HD.^1^ However, the Norwegian Cause of Death Registry reported a lower proportionate mortality attributable to suicide of 2.3% in patients with Huntington's disease;^3^ this is still double the approximately 1.2% suicide mortality in the general population.^30^

Little is known about risk factors for suicide attempts and completed suicide in Huntington's disease. Earlier studies showed that HDGECs with suicidal ideation are more often depressed, anxious or aggressive/irritable, and use more psychotropic medication.^5,24,31^ It is known from the general population that suicidal behaviour is associated with feelings of entrapment that result in an attempt to escape from an unbearable situation;^32^ having an incurable, progressive neurodegenerative disease may cause such a feeling of entrapment. The prospect of a loss of independence or having mental or neurocognitive problems may accelerate the transition from suicidal ideation to suicidal acts.

The key strength of our study is the substantial observation period (53 390 person-years) on which the analyses here are based, affording confidence in the robustness of the estimates generated. Other strengths are the broad representation of the Huntington's disease population in our sample, including extensive age and disease-stage coverage; the high-quality monitored data and the rigorous review and screening process that was applied to evaluate and categorise identified events; and the suicidal attempts estimates that encompass a conservative or more liberal range.

Our study also has some limitations. First, although Enroll-HD aims to recruit a large and population-representative sample, it is likely that there is a degree of selection bias; for instance, patients with Huntington's disease with severe impairments or severe psychiatric disorders may be less likely to participate. It is also possible that HDGECs who do not participate in Enroll-HD have other characteristics that are associated with risk of suicide attempt or suicide, such as people who are ashamed of their gene status or have difficulties in coping with the disease. Further, those participating in Enroll-HD may have unusually positive expectations of research and the development of effective therapeutics relative to those outside of the study. Second, per study protocol, individuals under 18 years of age are only able to participate if they have clinically diagnosed features of Huntington's disease and a confirmatory genetic test, restricting our sample of HDGECs in this age bracket. There is also limited ethnic diversity in Enroll-HD; Huntington's disease is principally a disease of Caucasian ancestry, and typically less prevalent in other ancestral groups, although there are exceptions. Another limitation is that most clinical characteristics were determined at the Enroll-HD visit before any events, as opposed to at the time of these events. Furthermore, lifetime prevalence of suicidal ideation may be underestimated as only one item of the C-SSRS was used, and reports of suicidal attempts may have been affected by recall bias or denial. It is also known that participant disclosure of information about suicidal ideation or behaviour is influenced by the associated stigma and therefore underreported.^33^ Given our reliance on reporting of events by participant or companion/caregiver, it is possible our incidence calculations are underestimated. Finally, it is likely that our incidence values specifically for 2019 may be underestimated, given the observed delay in event reporting.

The incidence of completed suicide and suicide attempts are greatly elevated in the Huntington's disease population, and this will be a critical feature of the disease until we have effective treatments. Our incidence estimates, based on a very large, prospectively assessed cohort, may serve as a reference for the Huntington's disease therapeutics community to assist in drug safety profile evaluation and, as therapeutic interventions become available, these metrics may also be valuable in assessing progress in clinical care. Future research may wish to leverage the comprehensive Enroll-HD dataset to investigate factors associated with suicide and suicidal attempt.

## Data Availability

Data available on request due to privacy/ethical restrictions. The data that support the findings of this study are available on request (specified dataset request; SPS) pending review by the Enroll-HD Scientific Review Committee; https://enroll-hd.org/forresearchers/access-data/.

## Supplementary material

For supplementary material accompanying this paper visit http://dx.doi.org/10.1192/bjo.2021.969. click here to view supplementary material

## References

[1] Brogueira Rodrigues F, Abreu D, Damásio J, Goncalves N, Correia-Guedes L, Coelho M, Survival, mortality, causes and places of death in a European Huntington's disease prospective cohort. Mov Disord Clin Pract 2017; 4: 737–42. 30363513 10.1002/mdc3.12502 PMC6174515

[2] Kachian ZR, Cohen-Zimerman S, Bega D, Gordon B, Grafman J. Suicidal ideation and behavior in Huntington's disease: systematic review and recommendations. J Affect Disord 2019; 250: 319–29. 30875675 10.1016/j.jad.2019.03.043

[3] Solberg OK, Filkuková P, Frich JC, Feragen KJB. Age at death and cause of death in patients with Huntington's disease in Norway. J Huntingtons Dis 2018; 7: 77–86. 29480207 10.3233/JHD-170270 PMC5870025

[4] Hubers AA, Reedeker N, Giltay EJ, Roos RAC, van Duijn E, van der Mast RC. Suicidality in Huntington's disease. J Affect Disord 2012; 136: 550–7. 22119091 10.1016/j.jad.2011.10.031

[5] van Duijn E, Vrijmoeth EM, Giltay EJ, Landwehrmeyer B, REGISTRY investigators of the European Huntington's Disease Network. Suicidal ideation and suicidal behavior according to the C-SSRS in a European cohort of Huntington's disease gene expansion carriers. J Affect Disord 2018; 228: 194–204. 29253686 10.1016/j.jad.2017.11.074

[6] Erlangsen A, Stenager E, Conwell Y, Andersen PK, Hawton K, Benros ME, Association between neurological disorders and death by suicide in Denmark. JAMA 2020; 323: 444–54. 32016308 10.1001/jama.2019.21834 PMC7042859

[7] Walker FO. Huntington's disease. Lancet 2007; 369: 218–28. 17240289 10.1016/S0140-6736(07)60111-1

[8] Keum JW, Shin A, Gillis T, Mysore JS, Abu Elneel K, Lucente D, The HTT CAG-expansion mutation determines age at death but not disease duration in Huntington disease. Am J Hum Genet 2016; 98: 287–98. 26849111 10.1016/j.ajhg.2015.12.018 PMC4746370

[9] van Duijn E, Craufurd D, Hubers AAM, Giltay EJ, Bonelli R, Rickards H, Neuropsychiatric symptoms in a European Huntington's disease cohort (REGISTRY). J Neurol Neurosurg Psychiatry 2014; 85: 1411–8. 24828898 10.1136/jnnp-2013-307343

[10] Turecki G, Brent DA. Suicide and suicidal behavior. Lancet 2016; 387: 1227–39. 26385066 10.1016/S0140-6736(15)00234-2 PMC5319859

[11] Huntington Study Group. Tetrabenazine as antichorea therapy in Huntington disease: a randomized controlled trial. Neurology 2006; 66: 366–72. 16476934 10.1212/01.wnl.0000198586.85250.13

[12] Dorsey ER, Brocht AF, Nichols PE, Darwin KC, Anderson KE, Beck CA, Depressed mood and suicidality in individuals exposed to tetrabenazine in a large Huntington disease observational study. J Huntingtons Dis 2013; 2: 509–15. 25062735 10.3233/JHD-130071

[13] Sampaio C, Ware JJ, Macleod M, Wagenmakers EJ, Munafò M. Reader response: evaluating depression and suicidality in tetrabenazine users with Huntington disease. Neurology 2019; 92: 447–8. 10.1212/WNL.0000000000006999 30804061

[14] Schultz JL, Killoran A, Nopoulos PC, Chabal CC, Moser DJ, Kamholz JA. Evaluating depression and suicidality in tetrabenazine users with Huntington disease. Neurology 2018; 91: e202–7. 29925548 10.1212/WNL.0000000000005817

[15] Sathe S, Ware J, Levey J, Neacy E, Blumenstein R, Muhlback A. Enroll-HD: an integrated clinical research platform and worldwide observational study for Huntington's disease. Front Neurol 2021; 12: 1190. 10.3389/fneur.2021.667420 PMC8416308 34484094

[16] Landwehrmeyer GB, Fitzer-Attas CJ, Giuliano JD, Gonçalves N, Anderson KE, Cardoso F, Data analytics from Enroll-HD, a global clinical research platform for Huntington's disease. Mov Disord Clin Pract 2016; 4: 212–24. 30363395 10.1002/mdc3.12388 PMC6174428

[17] Posner K, Brown GK, Stanley B, Brent DA, Yershova KV, Oquendo MA, The Columbia-Suicide Severity Rating Scale: initial validity and internal consistency findings from three multisite studies with adolescents and adults. Am J Psychiatry 2011; 168: 1266–77. 22193671 10.1176/appi.ajp.2011.10111704 PMC3893686

[18] Zhang Y, Long JD, Mills JA, Warner JH, Lu W, Paulsen JS, Indexing disease progression at study entry with individuals at-risk for Huntington disease. Am J Med Genet B Neuropsychiatr Genet 2011; 156B: 751–63. 21858921 10.1002/ajmg.b.31232 PMC3174494

[19] Mantalos P, Zografos K. Interval estimation for a binomial proportion: a bootstrap approach. J Stat Comput Simul 2008; 78: 1249–63.

[20] Center for Drug Evaluation and Research. Guidance for Industry: Suicidal Ideation and Behavior: Prospective Assessment of Occurrence in Clinical Trials. US Food and Drug Administration, 2012 (https://www.fda.gov/regulatory-information/search-fda-guidance-documents/guidance-industry-suicidal-ideation-and-behavior-prospective-assessment-occurrence-clinical-trials).

[21] Borges G, Nock MK, Haro Abad JM, Hwang I, Sampson NA, Alonso J, Twelve-month prevalence of and risk factors for suicide attempts in the World Health Organization World Mental Health Surveys. J Clin Psychiatry 2010; 71: 1617–28. 20816034 10.4088/JCP.08m04967blu PMC3000886

[22] Anderson KE, Eberly S, Groves M, Kayson E, Marder K, Young AB, Risk factors for suicidal ideation in people at risk for Huntington's disease. J Huntingtons Dis 2016; 5: 389–94. 27983561 10.3233/JHD-160206

[23] Honrath P, Dogan I, Wudarczyk O, Görlich KS, Votinov M, Werner CJ, Risk factors of suicidal ideation in Huntington's disease: literature review and data from Enroll-HD. J Neurol 2018; 265: 2548–61. 30167880 10.1007/s00415-018-9013-6

[24] Fiedorowicz JG, Mills JA, Ruggle A, Langbehn D, Paulsen JS; PREDICT-HD Investigators of the Huntington Study Group. Suicidal behavior in prodromal Huntington disease. Neurodegener Dis 2011; 8: 483–90. 21659725 10.1159/000327754 PMC3186721

[25] McGarry A, McDermott MP, Kieburtz K, Fung WLA, McCusker E, Peng J, Risk factors for suicidality in Huntington disease: an analysis of the 2CARE clinical trial. Neurology 2019; 92: e1643–51. 30850442 10.1212/WNL.0000000000007244 PMC6448454

[26] Reilmann R, McGarry A, Grachev ID, Savola JM, Borowsky B, Eyal E, Safety and efficacy of pridopidine in patients with Huntington's disease (PRIDE-HD): a phase 2, randomised, placebo-controlled, multicentre, dose-ranging study. Lancet Neurol 2019; 18: 165–76. 30563778 10.1016/S1474-4422(18)30391-0

[27] World Health Organization (WHO). Global Health Observatory Data: Suicide Rates, 2016. WHO, 2016 (https://www.who.int/gho/mental_health/suicide_rates/en/).

[28] Nordentoft M, Erlangsen A. Suicide, turning the tide. Science 2019; 365: 725. 31439766 10.1126/science.aaz1568

[29] Sørensen SA, Fenger K. Causes of death in patients with Huntington's disease and in unaffected first degree relatives. J Med Genet 1992; 29: 911–4. 1479606 10.1136/jmg.29.12.911 PMC1016212

[30] Statistics Netherlands. Ranglijst doodsoorzaken op basis van sterfte. [Ranking of Causes of Death Based on Mortality.] National Institute for Health and Environment, 2019 (https://www.volksgezondheidenzorg.info/ranglijst/ranglijst-doodsoorzaken-op-basis-van-sterfte).

[31] Hubers AA, van Duijn E, Roos RA, Craufurd D, Rickards H, Landwehrmeyer B, Suicidal ideation in a European Huntington's disease population. J Affect Disord 2013; 151: 248–58. 23876196 10.1016/j.jad.2013.06.001

[32] O'Connor RC, Kirtley OJ. The integrated motivational-volitional model of suicidal behaviour. Philos Trans R Soc Lond B Biol Sci 2018; 373: 20170268. 30012735 10.1098/rstb.2017.0268 PMC6053985

[33] Klonsky ED, May AM, Saffer BY. Suicide, suicide attempts, and suicidal ideation. Annu Rev Clin Psychol 2016; 12: 307–30. 26772209 10.1146/annurev-clinpsy-021815-093204

