# Supplementary material for: Incidence of completed suicide and suicide attempts in a global prospective study of Huntington's disease
Source: BJPsych Open. 2021 Aug 31;7(5):e158. doi: 10.1192/bjo.2021.969 (PMC8444051; doi:10.1192/bjo.2021.969)
Supplement: Supplementary file 1 [file S2056472421009698sup001.docx]

**Supplementary Text S1.** Imputation of missing or incomplete event dates.

Missing or incomplete event dates were imputed according to the following rules:

- If the event date was missing in full (i.e., month, day, and year = missing): the date on which the *Reportable Event* report was completed (*report*MM/*report*DD/*report*YY) was used as the imputed date.
- If the event date featured year only (*event*YY), and this was equivalent to the year in which the *Reportable Event* report was completed (*report*YY): the imputed event date was the date on which the *Reportable Event* report was completed: *report*MM/*report*DD/*report*YY.
- If the event date featured year only (*event*YY), and this was different to the year in which the *Reportable Event* form was completed (*report*YY): the imputed event date was as follows: 12/31/*event*YY.
- If the event date featured month and year only (*event*MM/*event*YY), and this was equivalent to the month and year in which the *Reportable Event* form was completed (*report*MM/*report*YY): the imputed event date was the date on which the *Reportable Event* form was completed: *report*MM/*report*DD/*report*YY.
- If the event date featured month and year only (*event*MM/*event*YY), and this was different to the month and year in which the *Reportable Event* form was completed (*report*MM/*report*YY): the imputed event date was as follows: *event*MM/15/*event*YY.
